# Differential Effects of 1α,25-Dihydroxyvitamin D_3_ on the Expressions and Functions of Hepatic CYP and UGT Enzymes and Its Pharmacokinetic Consequences In Vivo

**DOI:** 10.3390/pharmaceutics12111129

**Published:** 2020-11-23

**Authors:** Trang Nguyen Kieu Doan, Dang-Khoa Vo, Hyojung Kim, Anusha Balla, Yunjong Lee, In-Soo Yoon, Han-Joo Maeng

**Affiliations:** 1 Department of Pharmacy, College of Pharmacy, Gachon University, Incheon 21936, Korea; doannguyenkieutrang611@gmail.com (T.N.K.D.); vodangkhoa135@gmail.com (D.-K.V.); aanushaballa@gmail.com (A.B.); 2 Department of Pharmacology, Sungkyunkwan University School of Medicine, Suwon 16419, Korea; hjung93@skku.edu (H.K.); ylee69@skku.edu (Y.L.); 3 Department of Manufacturing Pharmacy, College of Pharmacy, Pusan National University, Busan 46241, Korea

**Keywords:** CYP, UGT, 1,25(OH)_2_D_3_, pharmacokinetics, metabolic kinetics, vitamin D receptor, drug-drug interactions

## Abstract

The compound 1α,25-Dihydroxyvitamin D_3_ (1,25(OH)_2_D_3_) is the active form of vitamin D_3_ and a representative ligand of the vitamin D receptor (VDR). Previous studies have described the impacts of 1,25(OH)_2_D_3_ on a small number of cytochrome P450 (CYP) and uridine diphosphate-glucuronyltransferase (UGT) enzymes, but comparatively little is known about interactions between several important CYP and UGT isoforms and 1,25(OH)_2_D_3_ in vitro and/or in vivo. Thus, we investigated the effects of 1,25(OH)_2_D_3_ on the gene and protein expressions and functional activities of selected CYPs and UGTs and their impacts on drug pharmacokinetics in rats. The mRNA/protein expressions of Cyp2b1 and Cyp2c11 were downregulated in rat liver by 1,25(OH)_2_D_3_. Consistently, the in vitro metabolic kinetics (V_max_ and CL_int_) of BUP (bupropion; a Cyp2b1 substrate) and TOL (tolbutamide; a Cyp2c11 substrate) were significantly changed by 1,25(OH)_2_D_3_ treatment in liver microsomes, but the kinetics of acetaminophen (an Ugt1a6/1a7/1a8 substrate) remained unaffected, consistent with Western blotting data for Ugt1a6. In rat pharmacokinetic studies, the total body clearance (CL) and nonrenal clearance (CL_NR_) of BUP were significantly reduced by 1,25(OH)_2_D_3_, but unexpectedly, the total area under the plasma concentration versus time curve from time zero to infinity (AUC) of hydroxybupropion (HBUP) was increased probably due to a marked reduction in the renal clearance (CL_R_) of HBUP. Additionally, the AUC, CL, and CL_NR_ for TOL and the AUC for 4-hydroxytolbutamide (HTOL) were unaffected by 1,25(OH)_2_D_3_ in vivo. Discrepancies between observed in vitro metabolic activity and in vivo pharmacokinetics of TOL were possibly due to a greater apparent distribution volume at the steady-state (V_ss_) and lower plasma protein binding in 1,25(OH)_2_D_3_-treated rats. Our results suggest possible drug-drug and drug-nutrient interactions and provide additional information concerning safe drug combinations and dosing regimens for patients taking VDR ligand drugs including 1,25(OH)_2_D_3_.

## 1. Introduction

Vitamin D_3_ is synthesized from 7-dehydroxycholesterol in the skin or acquired from diet [1], and is sequentially metabolized through two hydroxylation steps in the liver and kidney to 1α,25-dihydroxyvitamin D_3_ (1,25(OH)_2_D_3_, calcitriol), the active metabolite [2]. Further, 1,25(OH)_2_D_3_ regulates calcium and phosphorus homeostasis in mineral-regulating organs, such as the intestine, bone, kidney, and parathyroid glands by binding to vitamin D receptor (VDR) [3], and has been widely used to treat hypocalcemia, metabolic bone diseases, hypoparathyroidism, and cancer [2,4]. In addition, nonclinical studies reported that 1,25(OH)_2_D_3_ has several biological effects, which include antitumor [5], T-cell immunomodulatory [6], and neuroprotective effects [7].

Several studies have addressed the regulatory effects of 1,25(OH)_2_D_3_ on the expression and/or function of various drug transporters and drug metabolizing enzymes [8]. For example, the mRNA/protein expressions and activities of multidrug resistance-associated protein 4 (MRP4), P-glycoprotein (P-gp), cytochrome P450 3A4 (CYP3A4), and multidrug resistance-associated protein 2 (MRP2) in Caco-2 cells were increased by 1,25(OH)_2_D_3_ treatment in vitro [9,10]. In terms of transporter-mediated drug-drug interactions (DDIs), 1,25(OH)_2_D_3_ significantly reduced the renal clearances of cefdinir and cefadroxil by down-regulating organic anion transporter 1 (OAT1) and organic anion transporter 3 (OAT3) expressions in rat kidney [11], but enhanced the oral absorption of adefovir dipivoxil by inducing MRP4 in rat intestine [12]. In the mice treated with 1,25(OH)_2_D_3,_ the pharmacokinetics of digoxin, a P-gp probe drug, was significantly affected in vivo, resulting in increased total clearance and renal clearance via regulation of P-gp by VDR activation [13].

Regarding the expressional and/or functional regulations of metabolic enzymes by activated VDR, most of previous studies have focused on the CYP3A. For example, the protein expression of Cyp3A23 was up-regulated selectively in rat small intestine, but not in liver [14]. The protein expression of Cyp3a1 and Cyp3a2 in the rat ileum slices exposed to 1,25(OH)_2_D_3_ was strongly induced [15]. Consistently, Chow et al. reported the dose-dependent increases of total Cyp3a protein in the rat duodenum and proximal jejunum, accompanied with an increased mRNA expression of hepatic Cyp3a9 by 1,25(OH)_2_D_3_ treatment [16]. In the Caco-2 cells pretreated with VDR ligand drugs containing 1,25(OH)_2_D_3,_ it was consistently reported the CYP3A4 protein expression level was significantly induced [9], which had been demonstrated that CYP3A4 expression was upregulated by 1,25(OH)_2_D_3_ via binding to the specific vitamin D response element (VDRE) [17]. In a recent study, we found the expression and function of intestinal and hepatic CYP3A were differentially changed by 1,25(OH)_2_D_3_ treatment in vivo, and that these changes had significant impacts on the oral absorption and disposition of buspirone (a CYP3A substrate drug) in rats [2]. Likewise, the co-administration of ergocalciferol and cholecalciferol markedly reduced the plasma levels of atorvastatin, a CYP3A4 substrate, and its active metabolites in human [18].

However, to the best of our knowledge, only a few observations were reported about other CYPs, rather than CYP3A. 1,25(OH)_2_D_3_ treatment has also been reported to induce the mRNA expressions of CYP2B6, CYP2C9, and CYP3A4 in human hepatocytes and the activity of CYP7A1 in mouse and human hepatocytes [19,20,21]. Furthermore, 1,25(OH)_2_D_3_ induced the transcriptions of uridine diphosphate-glucuronyltransferase (UGT) isoforms such as UGT1A8 and UGT1A10 in human intestinal cell lines such as LS180, Caco-2 and HCT-116, and thus, increased the systemic clearance of mycophenolic acid, a substrate of UGT1A8 and UGT1A10 [22].

Several studies have investigated the impacts of 1,25(OH)_2_D_3_ on limited CYPs and UGTs, but there is a need to further investigate interactions between some CYP and UGT isoforms and 1,25(OH)_2_D_3_ treatment in vitro and/or in vivo. Moreover, in terms of DDIs, seven CYPs, such as CYP2B6 and CYP2C9, and seven UGTs, including UGT1A1 and UGT1A6, are suggested to investigate metabolism mediated interactions for new drug candidates and new drugs by the United States (US) Food and Drug Administration (FDA) [23]. Indeed, VDR ligand drugs have been clinically used and developed with therapeutic indications for osteoporosis, secondary hyperparathyroidism, psoriasis, cancer, and autoimmune diseases [8]. Therefore, in the present study, we examined the effects of 1,25(OH)_2_D_3_ on the gene expressions and functional activities, including kinetic analysis for selected CYPs and UGTs, and its impact on in vivo drug pharmacokinetics in rats. To examine the functional activities of enzymes, the following model CYP substrates were selected for the in vitro metabolism study using rat liver microsomes (RLMs); bupropion (BUP) for Cyp2b1 [24], tolbutamide (TOL) for Cyp2c11 [25], and acetaminophen (ACET) for Ugt1a6, Ugt1a7, and Ugt1a8 [26].

## 2. Materials and Methods

### 2.1. Chemicals and Reagents

Briefly, 1,25(OH)_2_D_3_, ACET, acetaminophen glucuronide (AG), BUP, hydroxybupropion (HBUP), carbamazepine (CARB), diltiazem (DIL), theophylline (THEO), TOL, 4-hydroxytolbutamide (HTOL), and uridine diphosphate glucuronic acid (UDPGA) were purchased from Sigma-Aldrich Co. (St. Louis, MO, USA). Dihydronicotinamide adenine dinucleotide phosphate (NADPH) was purchased from Corning, Inc. (New York, NY, USA). All solvents used were of HPLC grade. The PrimeScriptTM 1st strand cDNA Synthesis Kit and SYBR^®^ Premix Ex TaqTM II ROX plus were purchased from Takara Bio, Inc. (Shiga, Japan). Forward and reverse primers for quantitative polymerase chain reaction (qPCR) experiment were synthesized by Bioneer Co. (Daejeon, Korea).

### 2.2. 1,25(OH)_2_D_3_ Treatment in Rats

Male Sprague-Dawley (SD) rats (8 weeks, 260–280 g) were purchased from Nara Biotech Co. (Seoul, Korea). Animals were maintained in cages under controlled conditions under a 12-h dark/light cycle with free access to food and tap water, acclimated for at least five days in the laboratory before experiments, and allocated to a control group or 1,25(OH)_2_D_3_-treated group. The 1,25(OH)_2_D_3_ dosing solution was prepared by diluting 1,25(OH)_2_D_3_ in ethanol with 5 mL of corn oil (2.56 nmol/mL of final concentration). The dosing solution for the control group was prepared but the 1,25(OH)_2_D_3_ was omitted. The dosing solutions were administered intraperitoneally to rats at a dose of 1 mL/kg/day (2.56 nmol/kg/day as 1,25(OH)_2_D_3_) over four consecutive days. On the fifth day, rats were used for pharmacokinetic study [2,11,12,16,27].

### 2.3. Liver Histology

A segment of liver was removed from control and 1,25(OH)_2_D_3_-treated rats, washed with PBS. After fixing the liver segment with 4% polyoxymethylene for 1 day, the liver segments were cut vertically into thin slices, followed by staining with hematoxylin and eosin (H&E) (Maxdiagnostics, Seoul, South Korea). The H&E-stained samples were examined under a light microscope (200×) (Olympus JP/IX70, Olympus Optical, Tokyo, Japan).

### 2.4. Serum Chemistry

Rat serum was obtained from whole blood on the fifth day after the 1,25(OH)_2_D_3_ treatment. The assay of total protein, albumin, serum glutamic oxaloacetic transaminase (sGOT), and serum glutamic pyruvic transaminase (sGPT) was performed by Green Cross Reference Laboratory (Seoul, Korea).

### 2.5. Preparation of Proteins and Western Blotting

For protein extraction, the rat liver tissues were homogenized in RIPA buffer (#89900, ThermoFisher Scientific, Waltham, MA, USA) with protease/phosphatase inhibitors using a Diax 900 homogenizer. The homogenized liver samples were incubated in ice for 30 min (for complete lysis) with vortexing every 5 min, and then centrifuged at 14,000× *g* for 30 min. The supernatants were collected and diluted by 10 times. The protein levels were quantified using the BCA Protein Assay Kit (#23227, ThermoFisher Scientific, Waltham, MA, USA) with bovine serum albumin (BSA) standards. Total protein lysates were subjected to Western blot analysis. Lysates were mixed with 2X Laemmli buffer (Bio-Rad) supplemented with β-mercaptoethanol (Bio-Rad) and boiled for 5 min. Proteins were separated by SDS-PAGE and transferred onto nitrocellulose membranes (0.45 µm, Bio-Rad; cat##162-0115) for immunoblotting. Immunoblotting was performed with the following antibodies: rabbit CYP2C11 antibody (#ab3571, Abcam, Cambridge, UK), rabbit UGT1A6 antibody (#ab157476, Abcam, Cambridge, UK) and mouse CYP2B1/B2 antibody (#sc-53244, Santa Cruz Biotechnology, Dallas, TX, USA) and HRP-conjugated β-actin mouse antibody (Sigma; cat#A3854; 1:10,000). The following secondary antibodies were used: HRP-conjugated goat antibody to mouse IgG (Genetex; cat# GTX-213111-01, 1:5000), HRP-conjugated goat antibody to rabbit IgG (Genetex; cat# GTX-213110-01; 1:5000). Then, the bands were visualized via chemiluminescence (#34577, ThermoFisher Scientific, Waltham, MA, USA). Densitometric analysis of the bands was performed using ImageJ software (NIH; rsb.info.nih.gov/ij).

### 2.6. Real-Time Quantitative Polymerase Chain Reaction

Collected livers were frozen immediately with liquid nitrogen and stored at −80 °C. Trizol reagent in RNAiso Plus (Takara Bio, Inc., Shiga, Japan) was used to extract RNA from 100-mg tissue homogenates according to the manufacturer’s protocol. After purities and total RNA concentrations extracted from liver samples were determined at a wavelength of 260/280 nm with a Nanodrop 2000c spectrophotometer (Thermo Scientific, Waltham, MA, USA), synthesis of cDNA was processed from 1 μg of total RNA using the following conditions: incubation at 50 °C for 1 h, inactivation at 95 °C for 5 min, and cooling to 4 °C. The synthesized cDNA was then subjected to qPCR assays using SYBR^®^ Premix Ex TagTM on Stratagene Mx3005P (Agilent Technologies, Boblingen, Germany) using the following conditions: 95 °C for 10 min and 40 cycles of 95 °C for 15 s and 55 °C for 30 s. A “Comparative Quantitation” mode was selected, and fold expressions were calculated using the delta-delta method 2^−(△△Ct)^. GAPDH was used as the internal reference gene for normalization. The forward and reverse primers used for qPCR analysis are listed in Supplementary Table S1 [2,25,28,29].

### 2.7. In Vitro Metabolic Study Using Rat Liver Microsomes (RLMs)

RLMs were prepared according to a previously described method [30]. Rat livers were homogenized in ice-cold microsomal buffer (0.154 M KCl and 1 mM EDTA in 50 mM Tris-HCl (pH 7.4)). Resulting homogenates were centrifuged at 10,000 × *g* for 30 min and supernatants were further centrifuged at 100,000× *g* for 90 min. RLMs were obtained by suspending microsomal pellets in microsomal buffer. Protein contents of RLMs were determined using Lowry reagent (Sigma Aldrich Co., St. Louis, MO, USA). RLMs were obtained independently from three different rats for each experimental group. For CYP activity assays, a mixture of RLMs (protein concentration 1 mg/mL), 1.2 mM NADPH, and 100 mM phosphate buffer (pH 7.4) was pre-incubated in a thermomixer for 5 min at 37 °C and at 200 opm. The metabolic reaction was initiated by adding a 2.5 μL aliquot of drug solution in methanol with DMSO (final concentration 1% MEOH with 0.1% DMSO) to the preincubated microsomal reaction mixture (total volume of 250 μL). A system control study using buspirone (1 μM) was first confirmed (data not shown). The concentration ranges used for model substrates (20–1000 μM for BUP; 20–2000 μM for TOL) were obtained from previous studies [31,32,33]. A preliminary study at a specific drug concentration was first performed to assess the linearity of metabolite formation rate and determine a suitable incubation time for each substrate (i.e., 30 min). For UGT activity assays, a microsomal incubation mixture comprised of RLMs (protein concentration 1 mg/mL), MgCl_2_ (1 mM), ACET solution in DMSO and 100 mM phosphate buffer solution (pH 7.4) was pre-incubated in thermal mixer for 5 min at 37 °C and at 200 opm, and then 100 mM of UDPGA in phosphate buffer solution was added to initiate the reaction. A substrate concentration range from 0.1 to 30 mM was selected based on previous studies [29,34], and an incubation time of 90 min was chosen after a preliminary study with 1 mM ACET. Sampling was conducted at 0 min and at the end of incubation by transferring 50 μL aliquot of microsomal reaction mixture to 1.5 mL microcentrifuge tubes containing 100 μL of ice-cold internal standard (IS) solution in methanol. Resultant mixtures were immediately vortex-mixed to terminate the enzymatic reaction and then centrifuged at 15,000× *g* for 15 min at 4 °C. Supernatants were analyzed by ultra-performance liquid chromatography method with diode array detection (UPLC-DAD) or liquid chromatography-tandem mass spectrometry (LC-MS/MS) to determine metabolite levels.

### 2.8. In Vitro Plasma Protein Binding Study

A rat plasma protein binding study was performed using the rapid equilibrium dialysis kit (RED, 8 KDa molecular weight cut-off, Thermo Fisher Scientific, Waltham, MA, USA) according to a manufacture’s protocol. The plasma containing drug (5 μg/mL) was placed into the sample chamber, and then dialysis buffer was added to the buffer chamber. Upon sealing the cover of the unit, incubation was applied for 4 h at 37 °C on a shaking water-bath. To determine percent unbound (f_up_, %) in plasma, the drug concentrations in the plasma and buffer samples were analyzed by LC-MS/MS.

### 2.9. In Vivo Pharmacokinetic Study in Rats

In vivo pharmacokinetic animal experiments were accomplished according to the Guide for the Care and Use of Laboratory Animals issued by the National Institute of Health, as described previously [2,10,11]. Before starting the animal studies, all experimental protocols were reviewed and approved by the Animal Care and Use Committee of Gachon University (Approval no. GIACUC-R2019020; approved on July 1st, 2019). SD male rats (8–9 weeks, 250–300 g, Nara Biotech Co., Seoul, South Korea) had free access to food and water, acclimatized and maintained in the room under a 12-h light/dark cycle for a week before the study. After pretreatment with 1,25(OH)_2_D_3_ for 4 continuous days, the rats in the control and 1,25(OH)_2_D_3_ groups were anesthetized with Zoletil^®^ (Vibrac, TX, USA) (10 mg/kg i.m.) and then the femoral vein and artery were cannulated with polyethylene tubing (Clay Adams, NJ, USA) for drug administration and blood sampling, respectively, as described previously [2,11,12]. The pharmacokinetic experiment was initiated by drug administration after the rats were recovered from anesthesia. In the pharmacokinetic study of BUP, BUP in saline was administered intravenously at 5 mg/kg to control and 1,25(OH)_2_D_3_ -treated rats. Approximately 100 μL of blood was collected via the femoral artery at 0, 1, 5, 15, 30, 60, 120, 180, 240, 360, 480, and 600 min after the drug administration. In pharmacokinetic study of TOL, TOL in vehicle (DMSO:PEG400:saline = 5:40:55, *v*/*v*/*v*) was administered intravenously at 2 mg/kg. Blood samples were collected at 0, 1, 5, 15, 30, 60, 90, 120, 180, 240, 360, and 480 min later and immediately centrifuged at 14,000× *g* for 15 min at 4 °C. For compensation of fluid loss, a same volume of saline was intravenously provided after each sample collection. Plasma was then separated from whole blood cells and stored at −20 °C for further analysis. Urine samples were collected 0–4, 4–8, and 8–24 h after drug administration. The collected urine samples were diluted with distilled deionized water (DDW) 20- fold prior to LC-MS/MS analysis [11,12]. For the tissue distribution study of TOL, we sacrificed rats at 8 h after intravenous injection of the drug, and several organs including liver, and kidney, brain, spleen, and heart were taken, as described previously [11]. The weighted tissues were homogenized on ice by an electric homogenizer following adding 2-fold volume of PBS. The homogenates were stored before LC-MS/MS analysis.

### 2.10. Sample Preparation

Calibration standards for plasma, diluted urine samples and tissue homogenate samples of parent drugs and metabolites were prepared by mixing 10 μL of standard working solution with 90 μL of blank rat plasma, blank diluted urine, or blank tissue homogenate. Then, 200-μL IS solution was added to 100-μL biological samples and vortex-mixed for 1 min. Following centrifugation at 14,000× *g* for 15 min at 4 °C, supernatants were analyzed by UPLC-DAD or LC-MS/MS.

### 2.11. UPLC-DAD Analysis

UPLC-DAD was conducted using an Agilent Technologies 1290 Infinity II UHPLC system (Agilent Technologies, Santa Clara, CA, USA) with an autosampler (G7167B), a flexible pump (G7104A), a multicolumn thermostat (MCT-G7116B) a DAD detector (G7117A), and a Luna Omega Polar C18 column (100 × 2.1 mm, 1.6 μm; Phenomenex, Torrance, CA, USA). The mobile phase was a mixture of 0.2% acetic acid (pH 3.8, solvent A) and acetonitrile (ACN) (solvent B). For ACET, the mobile phase was eluted using the following gradient program: 10 *v*/*v* % solvent B for 3.5 min, 10 to 30 *v*/*v* % solvent B over 0.5 min, 30 *v*/*v* % solvent B for 6 min, and 10 *v*/*v* % solvent B for 5 min. For the measurement of HTOL in microsomal study, the mobile phase was eluted using the following gradient program: 25 *v*/*v* % solvent B for 5.5 min; 25 to 35 *v*/*v* % solvent B over 1 min; 35 *v*/*v* % solvent B for 8.5 min; and 25 *v*/*v* % solvent B for 5 min. For AG, the mobile phase consisted of 96 *v*/*v* % solvent A and 4 *v*/*v* % solvent B was elution isocratically for 19 min. ACET, AG, and HTOL were detected at 245, 245, and 230 nm, respectively. Rutin, CARB, and THEO were used as ISs for the analyses of ACET, HTOL, and AG, respectively. The flow rate used was 1 mL/min, and the sample injection volume was 5 μL for all the analytes except AG (10 μL).

### 2.12. LC-MS/MS Analysis

Quantitative determinations of BUP, HBUP, TOL and HTOL in pharmacokinetic studies samples were performed using an Agilent 6490 Triple Quadrupole LC/MS coupled with an Agilent Technologies 1260 HPLC system (Agilent Technologies, Santa Clara, CA, USA). Separations were conducted using a Synergi Polar-RP column (150 × 2.0 mm, 4 μm, 80 Å; Phenomenex, Torrance, CA, USA) using a 0.1% formic acid in water (solvent A) and ACN (solvent B) as the mobile phase. The injection volume was 2 µL for all analytes. For BUP and HBUP, the gradient elution was performed at 0.2 mL/min, as follows: 20 to 70 *v*/*v* % solvent B over 8 min; 70 *v*/*v* % solvent B for 2 min; and from 70 to 20 *v*/*v* % solvent B over 5 min. For TOL and HTOL, an isocratic elution was performed using 40% solvent B. The mass transitions of BUP, HBUP, DIL, TOL, HTOL, and CARB monitored were: 240.2→184.1, 256.1→238.1, 415.2→178, 271.1→155.0, 287.1→89.1, and 237.1→194.2, respectively.

### 2.13. Pharmacokinetic Analysis

In vitro K_m_ and V_max_ for metabolic reactions in RLMs were calculated based on the Michaelis-Menten equation using Sigma Plot software (Jandel Scientific, San Rafael, CA, USA). CL_int_ was calculated by dividing the V_max_ by the K_m_. Percentage (%) of free form was calculated by the concentration of buffer chamber by dividing the concentration of plasma chamber in the protein binding study. The following pharmacokinetic parameters were determined by non-compartmental analysis using WinNonlin^®^ 8.3 (Pharsight Co., Mountain View, CA, USA): total area under the plasma concentration versus time curve from time zero to infinity (AUC); total body clearance (CL), elimination half-life (t_1/2_); apparent distribution volume at the steady-state (V_ss_); the first moment of AUC (AUMC); and the mean residence time (MRT). CL_R_ was obtained by dividing the accumulated drug amount excreted in urine over 24 h by AUC [11], assuming the urinary recovery of the drug was completed 24 h after drug administration. Moreover, CL_NR_ was calculated to subtract CL_R_ from CL.

### 2.14. Statistical Analysis

*p*-values of <0.05 were considered to be statistically significant as determined by the two-tailed Student *t*-test between unpaired means for control and treatment groups. Results are presented as means ± standard deviation (SD)s, except T_max_ values, which are expressed as medians (ranges).

## 3. Results

### 3.1. Effects of 1,25(OH)_2_D_3_ on Liver Histology, Serum Chemistry and on The mRNA/Protein Expression Levels of Hepatic Cyps and Ugts in Rats

**Figure 1:**
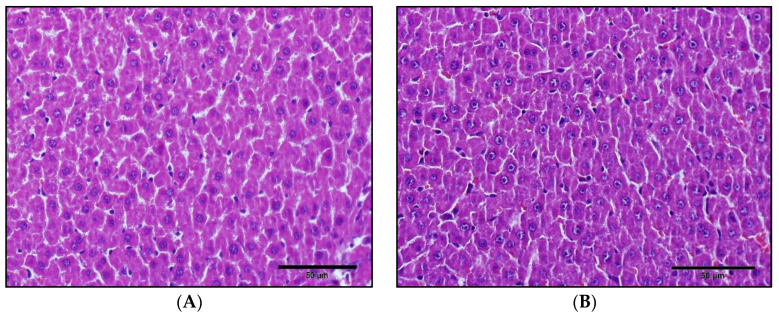
Representative histological sections of liver segments stained with hematoxylin and eosin in control (**A**) and 1α,25-dihydroxyvitamin D_3_ (1,25(OH)_2_D_3_)-treated (**B**) rats. The scale bars indicate 50 μm.

Initially, to investigate the possibility of liver toxicity, we examined liver histologies after four days of 1,25(OH)_2_D_3_ treatment. Histological sections of liver segments stained with hematoxylin and eosin in control and 1,25(OH)_2_D_3_-treated rats are shown in Figure 1. In these two groups, there was no apparent evidence of damage to liver tissue, such as inflammatory cell aggregation, disturbed hepatic architecture, vascular congestion, or necrosis. The number of cell nuclei is not different between two groups, whereas nucleoli were more distinctly shown in 1,25(OH)_2_D_3_-treated group. Moreover, when we compared the total plasma protein, plasma albumin, sGOT and sGPT between control and 1,25(OH)_2_D_3_-treated groups, no significant change was observed as shown in Table 1, indicating liver toxicity is unlikely by 1,25(OH)_2_D_3_ treatment in this study.

**Figure 2:**
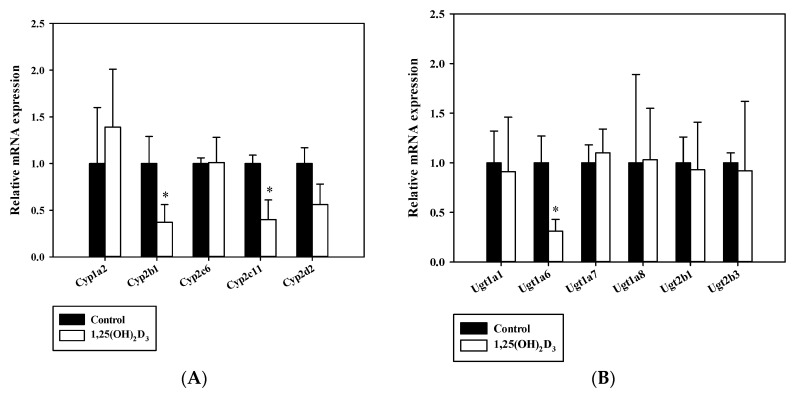
Comparison of mRNA expression levels of hepatic cytochrome P450 (CYP) (**A**) and uridine diphosphate-glucuronyltransferase (UGT) (**B**) enzymes in control rats (closed bars) and 1,25(OH)_2_D_3_-treated rats (open bars). The rectangular bars and their error bars represent means and standard deviations (*n* = 4–5). The asterisks indicate statistically significant differences compared to the control group (* *p* < 0.05).

**Figure 3:**
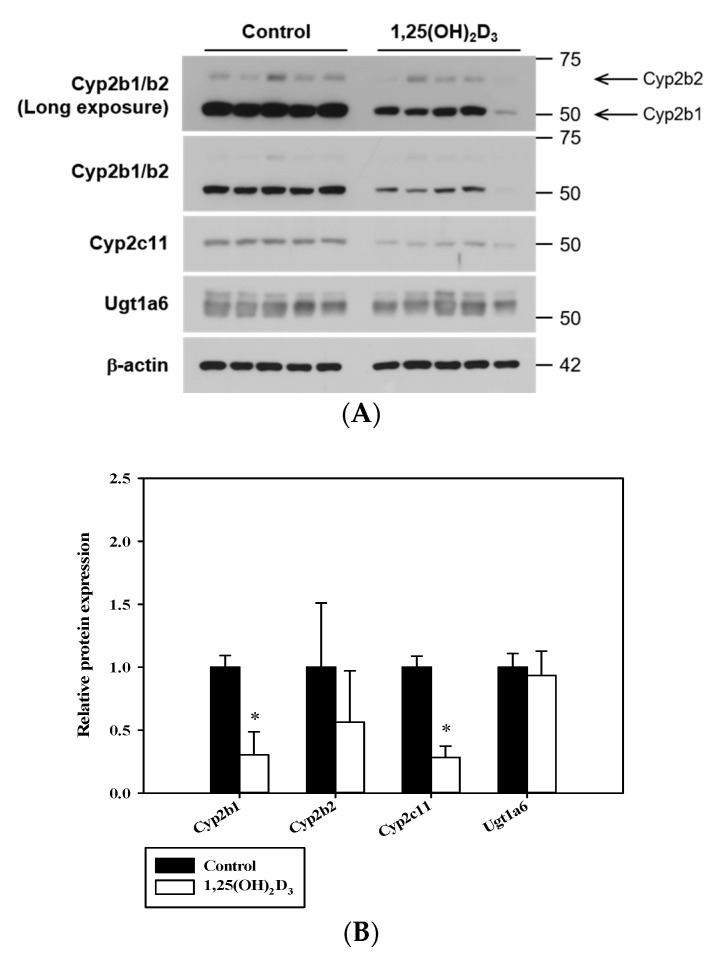
Comparison of protein expression levels of hepatic Cyp2b1/2b2, Cyp2c11 and Ugt1a6. (**A**) Western blot analysis for hepatic Cyp2b1/2b2, Cyp2c11 and Ugt1a6 in control rats and 1,25(OH)_2_D_3_-treated rats. (**B**) Densitometry of Western blotting for hepatic Cyp2b1/2b2, Cyp2c11 and Ugt1a6. The rectangular bars and their error bars represent means and standard deviations (*n* = 5). The asterisks indicate statistically significant differences compared to the control group (* *p* < 0.05).

The mRNA expressional regulations of hepatic CYPs containing Cyp1a2, Cyp2b1, Cyp2c6, Cyp2c11 and Cyp2d2 and UGTs such as Ugt1a1, Ugt1a6, Ugt1a7, Ugt1a8, Ugt2b1 and Ugt2b3 caused by VDR activation were investigated by quantitative real-time PCR (Figure 2). The mRNA expression levels of hepatic Cyp2b1 (*p* = 0.006), Cyp2c11 (*p* = 0.001) (Figure 2A), and Ugt1a6 in 1,25(OH)_2_D_3_-treated rats were significantly lower than in control rats (Figure 2B). However, no significant differences in the mRNA expressions of hepatic Cyp1a2, Cyp2c6, Cyp2d2, Ugt1a1, Ugt1a7, Ugt1a8, Ugt2b1, and Ugt2b3 were observed between the two groups (Figure 2). Moreover, hepatic Cyp2b1 protein expression was significantly decreased in 1,25(OH)_2_D_3_-treated rats (*p* = 0.0006) whereas Cyp2b2 protein expression was lower, but this difference is not significant (*p* = 0.1728) (Figure 3). Hepatic Cyp2c11 protein level was also significantly lower in 1,25(OH)_2_D_3_-treated rats compared with the control group (*p* = 0.0001). However, hepatic Ugt1a6 protein expression remained unchanged in 1,25 (OH)_2_D_3_-treated rats (*p* = 0.5192) (Figure 3).

### 3.2. Effects of 1,25(OH)_2_D_3_ on the Functional Activities of CYPs and UGTs in RLMs

**Figure 4:**
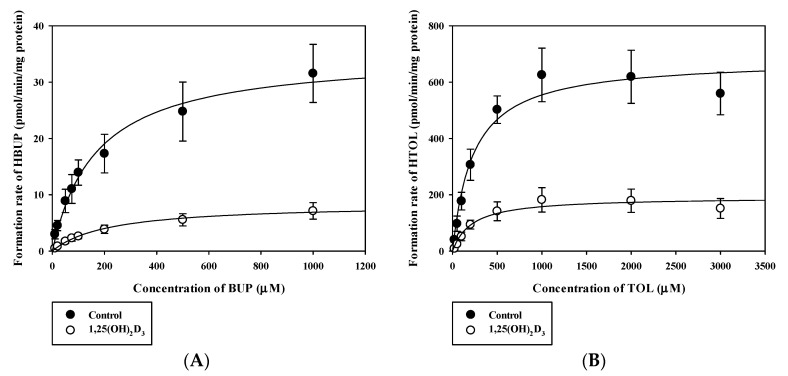
Mean velocities of formation of HBUP (**A**; bupropion hydroxylation), and HTOL (**B**; tolbutamide 4-hydroxylation) in rat liver microsomes (RLMs) obtained from the control (closed circle) and the 1,25(OH)_2_D_3_-treated rat group (open circle). The circles and error bars represent means and standard deviations, respectively (*n* = 3).

**Figure 5:**
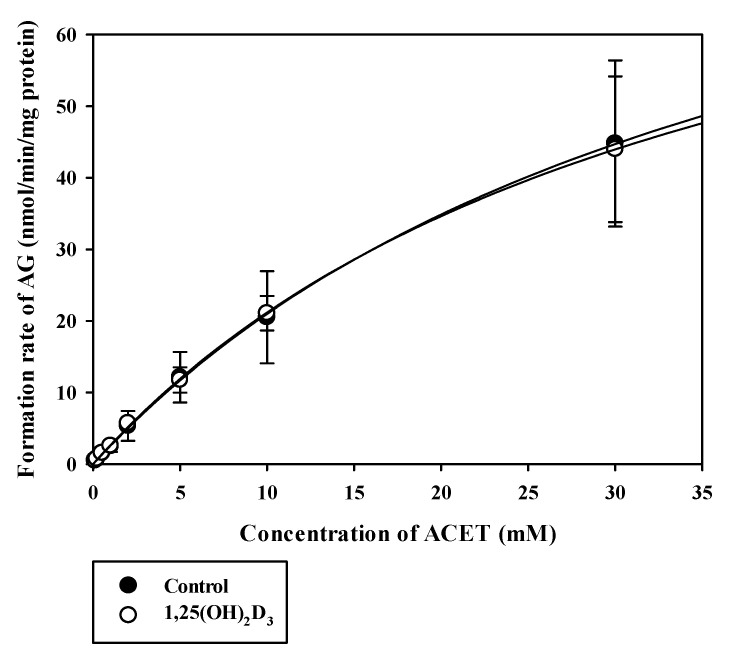
Mean velocities of formation of AG (acetaminophen glucuronidation) in RLMs obtained from the control (closed circle) and the 1,25(OH)_2_D_3_-treated rat group (open circle). The circles and error bars represent means and standard deviations, respectively (*n* = 3).

Using RLMs, we continued to investigate whether significant expressional changes of Cyp2b1, Cyp2c11, and Ugt1a6 by 1,25(OH)_2_D_3_ affected metabolic activity in vitro or not. Concentration dependencies of the metabolic activities (i.e., metabolite formation rates) of BUP, TOL, and ACET using RLMs from control and 1,25(OH)_2_D_3_ -treated rats were observed to determine the formed metabolites, HBUP, HTOL, and AG, as shown in Figure 4 and Figure 5.

Calculated K_m_, V_max_, and CL_int_ values for BUP hydroxylation (Cyp2b1), TOL hydroxylation (Cyp2c11), and ACET glucuronidation (Ugt1a6) in control and 1,25(OH)_2_D_3_-treated rat liver microsomes are listed in Table 2 and Table 3, respectively. Notably, V_max_ values for the metabolism of both BUP and TOL were significantly lower in 1,25(OH)_2_D_3_-treated rats than in controls (*p* = 0.0025 for BUP and 0.0012 for TOL), consistent with quantitative real-time PCR data, but no significant intergroup difference was observed for K_m_ values. Consequently, calculated CL_int_ values were significantly lower in 1,25(OH)_2_D_3_-treated rats than in controls (by 82%, *p* = 0.0075 for BUP and by 70%, 0.0051 for TOL) (Table 2). In contrast, no significant intergroup difference was observed for K_m_, V_max_, and CL_int_ values for the metabolism of ACET (Table 3).

### 3.3. Effects of 1,25(OH)_2_D_3_ on the Pharmacokinetics of BUP and TOL

**Figure 6:**
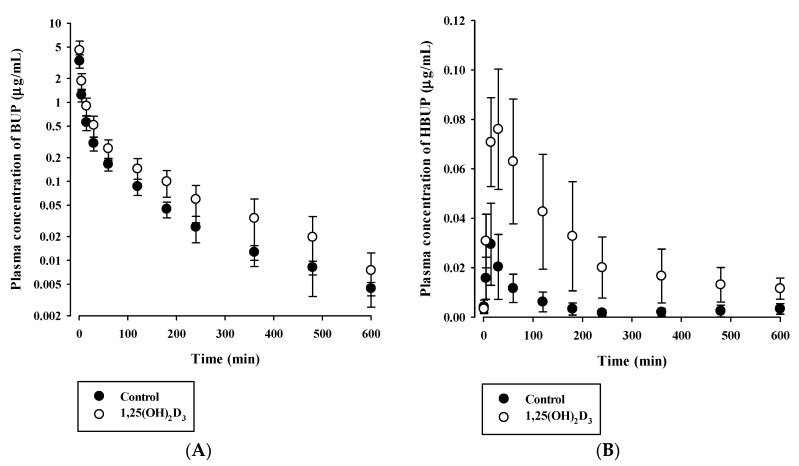
Plasma concentration versus time profiles of bupropion (BUP) (**A**) and the formed metabolite, hydroxybupropion (HBUP) (**B**) in control (closed circle) and 1,25(OH)_2_D_3_-treated rats (open circle) after intravenous administration of BUP at 5 mg/kg. The circles and error bars represent means and standard deviations, respectively (*n* = 6–7).

Since calculated CL_int_ values for BUP and TOL were found to be significantly changed in vitro, we further investigated the effects of 1,25(OH)_2_D_3_ on the pharmacokinetics of BUP (Cyp2b1 substrate) and TOL (Cyp2c11 substrate) in vivo. Plasma concentration–time curves of BUP and HBUP (formed metabolite) after intravenous administration of 5 mg/kg BUP in control and 1,25(OH)_2_D_3_ treated rats are presented in Figure 6. Plasma concentration levels of both BUP and HBUP were increased in the 1,25(OH)_2_D_3_ treated group, compared to control group. The pharmacokinetic parameters of BUP and its formed metabolite, HBUP, are provided in Table 4. Several obvious alterations were found in the pharmacokinetics of BUP in rats treated with 1,25(OH)_2_D_3_. In particular, the AUC of BUP was significantly higher by 60.7% (*p* < 0.001), as expected. CL and CL_NR_ were significantly lower by 34% and 34.8% (*p* = 0.0004 for CL and *p* = 0.0003 for CL_NR_), respectively, in 1,25(OH)_2_D_3_ -treated rats. However, 1,25(OH)_2_D_3_ -treated rats did not exhibit any significant change in MRT or terminal half-life versus controls. Notably, the AUC of HBUP and the AUC ratio between HBUP and BUP were significantly higher (*p* = 0.001 for AUC_HBUP_ and *p* = 0.015 for AUC_HBUP_/AUC_BUP_) and the CL_R_ of HBUP was significantly lower (*p* = 0.009) in 1,25(OH)_2_D_3_ -treated rats (Table 4).

**Figure 7:**
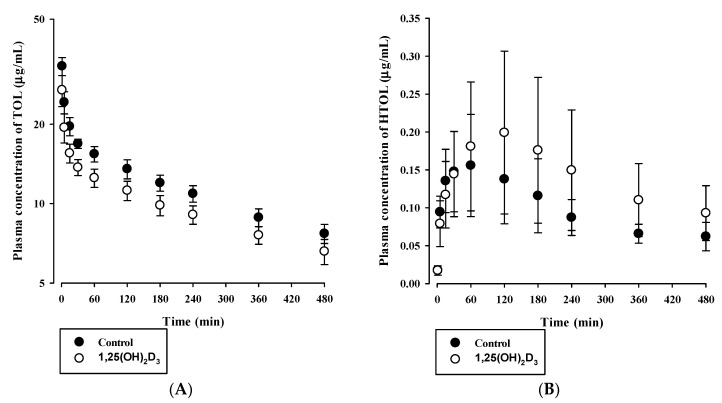
Plasma concentration versus time profiles of tolbutamide (TOL) (**A**) and the formed metabolite, 4-hydroxytolbutamide (HTOL) (**B**) in control (closed circle) and 1,25(OH)_2_D_3_-treated rats (open circle) after intravenous administration of TOL at 2 mg/kg. The circles and error bars represent means and standard deviations, respectively (*n* = 9–10).

In addition, the Figure 7 shows the plasma concentration–time profiles of TOL (Cyp2c11 substrate, Figure 7A) and HTOL (formed metabolite, Figure 7B) after intravenous administration of 2 mg/kg TOL to control and 1,25(OH)_2_D_3_-treated rats, respectively, and the calculated pharmacokinetic parameters are summarized in Table 5. No significant intergroup difference was found between the AUC values of TOL and HTOL (*p* = 0.563 for TOL and *p* = 0.0871 for HTOL). However, the CL_R_ values of TOL and HTOL were significantly lower in 1,25(OH)_2_D_3_-treated rats (*p* = 0.0405 for TOL and *p* = 0.00304 for HTOL). In addition, the V_ss_ of TOL in 1,25(OH)_2_D_3_-treated rats was significantly greater than that in control rats (*p* = 0.000807, Table 5).

Then, we further investigated tissue distribution of TOL and HTOL at the terminal phase (i.e., 8 h) after intravenous administration of TOL. Table 6 summarizes tissue to plasma concentration ratios (K_p_) of TOL and HTOL for liver, kidney, spleen, heart, and brain in both groups. To be consistent with the systemic pharmacokinetic result (i.e., increased V_ss_), the tissue to plasma concentration ratios of TOL in most of tissues such as liver, brain, spleen, and heart were significantly increased (*p* < 0.05), except the kidney, suggesting that tissue distribution of TOL was higher in 1,25(OH)_2_D_3_-treated rats. Similarly, the formed metabolite, HTOL, also showed significantly increased tissue to plasma concentration ratios for the kidney, spleen, and heart, compared to control group (*p* < 0.05).

In addition, when the percentage of free form (f_up_, %) for TOL was compared between control and 1,25(OH)_2_D_3_-treated group using rat plasma protein binding assay, f_up_ values were found to be 3.51 ± 0.73% and 13.1 ± 0.3% (*n* = 3 per group, *p* < 0.05), respectively. This result suggests that 1,25(OH)_2_D_3_-treatment may affect the plasma protein binding of TOL in rats, resulting in the increased V_ss_ in vivo consequently.

## 4. Discussion

In the present study, a rat model was chosen to investigate the effect of 1,25(OH)_2_D_3_ on hepatic CYPs and UGTs, due to the similar hepatic VDR distributions in human and rats [35,36]. In a recent study, we reported that 1,25(OH)_2_D_3_ treatment affects intravenous and oral pharmacokinetics of buspirone, a CYP3A substrate, in rats due to the differential regulation of hepatic and intestinal CYP3A metabolic activities. Likewise, we designed the present study to investigate if the regulating effect of 1,25(OH)_2_D_3_ (the active form of vitamin D) on the expressions and activities of hepatic CYPs and UGTs other than CYP3A, and its consequences on pharmacokinetics of the specific substrates in vivo. Among the five Cyps and six Ugts enzymes tested, the mRNA and/or protein expression levels of hepatic Cyp2b1, Cyp2c11, and Ugt1a6 were found to be significantly reduced by 1,25(OH)_2_D_3_, whereas those of other enzymes were unaltered (Figure 2 and Figure 3). The extent of change in protein levels for Cyp2b1 or Cyp2c11 coincided with its mRNA results.

The H&E staining data with serum chemistry data shows that the apparent liver damage by 1,25(OH)_2_D_3_ is unlikely (Figure 1 and Table 1). However, other effects on hepatocytes, such as nucleoli and lipid accumulation, by 1,25(OH)_2_D_3_ treatment still need to be investigated. For examples, although the effect of 1,25(OH)_2_D_3_ on hepatic lipid accumulation is inconclusive, a few previous studies have reported that 1,25(OH)_2_D_3_ -treatment reduces hepatic triglyceride accumulation in mice [37,38].

The in vitro microsomal metabolism study was conducted on Cyp2b1, Cyp2c11, and Ugt1a6, the mRNA expression levels of which were significantly altered by 1,25(OH)_2_D_3_ treatment. The V_max_ and CL_int_ for Cyp2b1-mediated BUP hydroxylation (i.e., formation rate) and Cyp2c11-mediated TOL hydroxylation (i.e., formation rate) were significantly reduced by 1,25(OH)_2_D_3_ treatment, which concurred with qPCR data. However, the enzyme kinetic parameters of ACET glucuronidation were comparable in the two groups. In SD rats, the formation of AG is known to be catalyzed by multiple UGTs (i.e., Ugt1a7, Ugt1a6, and Ugt1a8; listed in order of decreasing contribution to ACET glucuronidation) [26]. Although the mRNA expression level of Ugt1a6 was significantly lower, the protein level of Ugt1a6 remained unchanged by 1,25(OH)_2_D_3_ treatment. Collectively, the unaltered metabolic functional activity of ACET is consistent with the protein level of Ugt1a6, not mRNA level. Also, it might be attributed to the involvements of multiple UGTs, rather than Ugt1a6 alone, in the formation of AG.

Based on in vitro microsomal metabolism data, we investigated in vivo pharmacokinetic consequences for BUP and TOL, whose in vitro metabolite formation kinetics were significantly altered by 1,25(OH)_2_D_3_ treatment (Table 2). As the elimination of BUP mainly occurs through the hepatic route [31], the reduced total clearance of BUP can be explained mostly due to the decreased Cyp2b1-mediated BUP hydroxylation in 1,25(OH)_2_D_3_-treated rats. Moreover, renal clearance accounted for only a minor portion of total clearance (i.e., 0.7% in control rats and 1.92% in 1,25(OH)_2_D_3_-treated rats). As shown in Table 4, the increased AUC and decreased CL and CL_NR_ of BUP in 1,25(OH)_2_D_3_-treated rats agreed well with its decreased CL_int_ in RLMs. However, the AUC of HBUP and the AUC ratio of HBUP to BUP (AUC_HBUP_/AUC_BUP_) were significantly elevated by 1,25(OH)_2_D_3_, though BUP metabolism was reduced. This observation may occur due to markedly lower HBUP renal excretion (CL_R_) in 1,25(OH)_2_D_3_-treated rats and/or the involvement of other enzymes such as Cyp2c and Cyp3a in BUP metabolism [39], which requires further investigation. Since bupropion supplemented with vitamin D_3_ can be clinically used to help to lessen major depression [40] and VDR ligand drugs have been developed with various therapeutic indications [8], the further study is required regarding clinical relevance of this pharmacokinetic change in rats. However, in the case of TOL, the AUC, CL, and CL_NR_ of TOL, and the AUC of HTOL remained unchanged in vivo. This is inconsistent with the TOL metabolism obtained from in vitro microsomal study. As the CL_NR_ of TOL was very low (Table 5), it is plausible that TOL is a drug with a low hepatic extraction ratio. This suggests that the hepatic clearance of TOL depends on protein binding and intrinsic metabolic clearance. Thus, the unaltered AUC, CL, and CL_NR_ of TOL could be attributed to the overall net effect of the increased free fraction in plasma and decreased intrinsic clearance in 1,25(OH)_2_D_3-_treated rats. We also observed the significantly greater V_ss_ and lower CL_R_ of TOL in 1,25(OH)_2_D_3_-treated rats (Table 5). Consistently, significantly increased tissue distribution of TOL for liver, spleen, heart, and brain were observed with lower plasma protein binding of TOL in 1,25(OH)_2_D_3_-treated rats (Table 6). Namely, the increase in V_ss_ is likely to be caused by changes in protein binding of TOL by 1,25(OH)_2_D_3_-treatment. Also, changes of membrane transport in tissues may be considered as another possible mechanism. This topic warrants further study. Previous studies on the biological membrane transport of TOL reported that it was transported across Caco-2 cell monolayers by a pH-dependent system presumably shared with other organic anions such as benzoic acid [41], and that the transport of TOL across the blood-brain barrier by a non-P-glycoprotein-mediated efflux system was inhibited by sulfonamides [42]. Notably, the CL_R_ of HTOL was markedly reduced in 1,25(OH)_2_D_3_-treated rats even though kidney function was unaffected by treatment (Table 5) [11], which might have been responsible for the insignificant increase of AUC of HTOL. The mechanisms involved need further study.

Generally, the activities of hepatic CYP enzymes might be altered in patients taking VDR-ligand nutrients and drugs, which suggests the possibility of pharmacokinetic interactions between VDR ligands drugs and CYP substrates. Hence, the results of the present study predict possible drug-drug and drug-nutrient interactions and provide practical information on effective and safe drug combinations and dosing regimens for patients taking VDR ligand drugs such as 1,25(OH)_2_D_3_.

## 5. Conclusions

The current study shows the mRNA/protein expressions of Cyp2b1 and Cyp2c11, are downregulated by 1,25(OH)_2_D_3_ in rats. Furthermore, the in vitro metabolic kinetics (V_max_ and CL_int_) of BUP (a Cyp2b1 substrate) and TOL (a Cyp2c11 substrate) were significantly changed by treating RLMs with 1,25(OH)_2_D_3_, as indicated by qPCR and Western blotting results, whereas that of acetaminophen (an Ugt1a6/1a7/1a8 substrate) was unaffected, consistent with data from Western blot analysis. Rat pharmacokinetic studies showed the CL and CL_NR_ of BUP were significantly reduced by 1,25(OH)_2_D_3_ treatment, but that surprisingly, the AUC of HBUP was increased (probably due to the markedly reduced CL_R_ of HBUP). Nevertheless, the AUC, CL, and CL_NR_ of TOL and the AUC of HTOL remained unchanged in vivo. These discrepancies between in vitro metabolic activities and in vivo pharmacokinetics of TOL might be partially due to a greater V_ss_ and lower plasma protein binding in 1,25(OH)_2_D_3_-treated rats. To the best of our knowledge, the present study is the first to describe the impacts of 1,25(OH)_2_D_3_ on metabolic functions and systemic pharmacokinetics of BUP and TOL in rats.

## Figures and Tables

**Table 1:** Serum chemistry parameters for liver function obtained from control and 1,25(OH)_2_D_3_-treated rats (*n* = 3).

Parameters	Control	1,25(OH)_2_D_3_
Total protein (g/dL)	5.60 ± 0.30	5.73 ± 0.25
Albumin (g/dL)	3.73 ± 0.12	3.97 ± 0.15
sGOT (IU/L)	156 ± 16	158 ± 35
sGPT (IU/L)	22.3 ± 3.5	22.7 ± 3.2

sGOT, serum glutamine oxaloacetate transaminase; sGPT, serum glutamate pyruvate transaminase.

**Table 2:** Estimated in vitro V_max_, K_m_, and CL_int_ for metabolism of probe substrates for Cyp2b1 and Cyp2c11 in RLMs obtained from control and 1,25(OH)_2_D_3_-treated rats (*n* = 3).

Kinetic Parameters	BUP (Cyp2b1)		TOL (Cyp2c11)	
	Control	1,25(OH)_2_D_3_	Control	1,25(OH)_2_D_3_
V_max_^a^	35.20 ± 6.62	8.41 ± 1.76 *	683.37 ± 94.05	191.57 ± 44.01 *
K_m_^b^	171.70 ± 39.46	219.80 ± 29.37	232.83 ± 51.33	214.63 ± 23.83
CL_int_^c^	0.21 ± 0.06	0.038 ± 0.01 *	3.00 ± 0.63	0.89 ± 0.18 *

* Significantly different from the control group (*p* < 0.05); ^a^ pmol/min/mg protein for BUP and TOL; ^b^ μM for BUP and TOL; ^c^ μL/min/mg protein.

**Table 3:** Estimated in vitro V_max_, K_m_, and CL_int_ for metabolism of probe substrate for UGTs in RLMs obtained from control and 1,25(OH)_2_D_3_-treated rats (*n* = 3).

Kinetic Parameters	ACET (Ugt1a6/1a7/1a8)	
	Control	1,25(OH)_2_D_3_
V_max_^a^	104.04 ± 17.57	115.12 ± 75.10
K_m_^b^	41.06 ± 0.89	44.18 ± 34.79
CL_int_^c^	2.64 ± 0.89	2.77 ± 0.40

^a^ nmol/min/mg protein for ACET; ^b^ mM for ACET; ^c^ μL/min/mg protein.

**Table 4:** Pharmacokinetic parameters of BUP and HBUP after intravenous administration of 5 mg/kg BUP in control and 1,25(OH)_2_D_3_-treated rats (*n* = 6–7).

Pharmacokinetic Parameters	Control	1,25(OH)_2_D_3_
**BUP**		
AUC (μg·min/mL)	54.38 ± 7.65	96.31 ± 23.65 *
t_1/2_ (min)	155.4 ± 25.9	134.7 ± 48.70
CL (mL/min/kg)	93.64 ± 14.24	54.60 ± 13.37 *
CL_R_ (mL/min/kg)	0.662 ± 0.635	1.053 ± 0.407
CL_NR_ (mL/min/kg)	93.08 ± 13.73	53.55 ± 13.02 *
MRT (min)	71.44 ± 10.29	88.43 ± 19.63
V_ss_ (×10^3^ mL/kg)	7.815 ± 1.953	5.403 ± 1.867
Ae_0-24h_ (% of dose)	0.719 ± 0.613	1.791 ± 0.031 *
**HBUP**		
AUC (μg·min/mL)	4.610 ± 1.898	19.77 ± 9.04 *
t_1/2_ (min)	160.4 ± 37.6	197.0 ± 54.2
T_max_ (min)	15	15 (15–60)
C_max_ (µg/mL)	0.046 ± 0.032	0.068 ± 0.021
CL_R_ (mL/min/kg)	0.222 ± 0.124	0.077 ± 0.031 *
Ae_0-24h_ (% of dose)	0.015 ± 0.007	0.026 ± 0.008 *
AUC_HBUP_/AUC_BUP_	0.090 ± 0.065	0.209 ± 0.084 *

* Significantly different from the control group (*p* < 0.05).

**Table 5:** Pharmacokinetic parameters of TOL and HTOL after intravenous administration of 2 mg/kg TOL in control and 1,25(OH)_2_D_3_-treated rats (*n* = 9–10).

Pharmacokinetic Parameters	Control	1,25(OH)_2_D_3_
**TOL**		
AUC (×10^3^ μg·min/mL)	10.807 ± 1.877	10.184 ± 2.623
t_1/2_ (min)	462.7 ± 153.9	563.8 ± 207.5
CL (mL/min/kg)	0.189 ± 0.027	0.206 ± 0.045
CL_R_ (μL/min/kg)	0.208 ± 0.103	0.039 ± 0.015 *
CL_NR_ (mL/min/kg)	0.189 ± 0.030	0.206 ± 0.045
MRT (min)	657.4 ± 204.5	795.4 ± 290.5
V_ss_ (mL/kg)	119.9 ± 15.6	153.1 ± 19.5 *
Ae_0-24h_ (% of dose)	0.124 ± 0.059	0.024 ± 0.016 *
**HTOL**		
AUC (μg·min/mL)	100.8 ± 65.2	151.9 ± 52.0
t_1/2_ (min)	521.3 ± 499.0	629.7 ± 491.6
T_max_ (min)	60 (15–120)	120 (60–240)
C_max_ (µg/mL)	0.167 ± 0.067	0.207 ± 0.119
CL_R_ (mL/min/kg)	2.713 ± 0.570	0.873 ± 0.134 *
Ae_0-24h_ (% of dose)	9.356 ± 0.471	5.539 ± 2.305 *
AUC_HTOL_/AUC_TOL_	0.0097 ± 0.070	0.0155 ± 0.0059

* Significantly different from the control group (*p* < 0.05).

**Table 6:** Tissue to plasma concentration ratios (K_p_) of TOL and HTOL at 8 h after intravenous injection of 2 mg/kg TOL in control and 1,25(OH)_2_D_3_-treated rats (*n* = 4–6).

Organs	Control	1,25(OH)_2_D_3_
**TOL**		
Liver	0.133 ± 0.002	0.166 ± 0.019 *
Kidney	0.139 ± 0.008	0.154 ± 0.014
Brain	0.021 ± 0.001	0.030 ± 0.004 *
Spleen	0.169 ± 0.030	0.175 ± 0.045 *
Heart	0.120 ± 0.003	0.184 ± 0.036 *
**HTOL**		
Liver	0.831 ± 0.152	0.657 ± 0.129
Kidney	5.335 ± 1.127	7.768 ± 0.014 *
Brain	0.020 ± 0.008	0.019 ± 0.004
Spleen	0.137 ± 0.010	0.163 ± 0.008 *
Heart	0.175 ± 0.013	0.252 ± 0.036 *

* Significantly different from the control group (*p* < 0.05).

## Supplementary Materials

The following are available online at https://www.mdpi.com/1999-4923/12/11/1129/s1, Table S1: Forward and reverse primers used in qPCR analysis for various rat CYP and UGT enzymes.
