# Supplementary material for: Differential Effects of 1α,25-Dihydroxyvitamin D3 on the Expressions and Functions of Hepatic CYP and UGT Enzymes and Its Pharmacokinetic Consequences In Vivo
Source: Pharmaceutics. 2020 Nov 23;12(11):1129. doi: 10.3390/pharmaceutics12111129 (PMC7700423; doi:10.3390/pharmaceutics12111129)
Supplement: Supplementary file 1 [file pharmaceutics-12-01129-s001.pdf]

pharmaceutics

Supplemental Material: Differential Effects of 1α,25-Dihydroxyvitamin D3 on The Expressions and Functions of Hepatic CYP and UGT Enzymes and Its Pharmacokinetic Consequences in Vivo

Trang Nguyen Kieu Doan, Dang-Khoa Vo, Hyojung Kim, Anusha Balla, Yunjong Lee, In-Soo Yoon and Han-Joo Maeng

Table S1. Forward and reverse primers used in qPCR analysis for various rat CYP an d UGT enzymes [1–4].

Genes	Forward Primers	Reverse Primers	Product Size (bp)	Ref.
Cyp1a2	ACGTGAGCAAAGAGGCTAACCA	ATTAGCCACCGATTCCACCAC	104	[1]
Cyp2b1	CAAGGAGAGTGGCATTGGAAAA	AGGCCATTCCCAACAGAACTGGG	106	[1]
Cyp2c6	CTTCAAGATTCAAGAAATATCCA	GTGAATTACCAGTGCTACA	166	[2]
Cyp2c11	CGCACGGAGCTGTTTTTGTT	GCAAATGGCCAAATCCACTG	115	[1]
Cyp2d2	GCAAAGTCTTCCCCAAGCTCA	GGAAGGCATCAGTCATGTCTCG	114	[1]
Ugt1a1	TGTCCTACGTGCCCAAGAGTT	GTCAGGACTAAG AAGGTCCTTG	185	[3]
Ugt1a6	GATGGCTCCTCTAAGAGACTA	GATCACACCACAGGGCATGG	160	[3]
Ugt1a7	CAGACCCCGGTGACTATGACA	CAACGTGAAGTCTGTGCGTAACA	72	[3]
Ugt1a8	GAGGGCATGAGGTGGTGGTA	CACGGTAAAATTCAGCGACTTTC	71	[3]
Ugt2b1	AAAGGAGCTGCTGTTAGAGTTG	GAACCAGCTAAGGTCATGCAG	234	[3]
Ugt2b3	CTACAGATAAGTTGCTGTTTCCA	CATCTTTACTGACAGATGTAGGG	220	[3]
Gapdh	CGCTGGTGCTGAGTATGTCG	CTGTGGTCATGAGCCCTTCC	266	[4]

References

1. Kawase, A.; Fujii, A.; Negoro, A.; Akai, R. Differences in cytochrome P450 and nuclear receptor mRNA levels in liver and small intestines between SD and DA rats. *Drug Metab. Pharmacokinet*. **2008**, *23*, 196–206.
2. Fukuno, S.; Nagai, K.; Kasahara, K.; Mizobata, Y.; Omotani, S.; Hatsuda, Y.; Myotoku, M.; Konishi, H. Altered tolbutamide pharmacokinetics by a decrease in hepatic expression of CYP2C6/11 in rats pretreated with 5-fluorouracil. *Xenobiotica* **2018**, *48*, 53–59.
3. Alkharfy, K.M.; Poloyac, S.M.; Congiu, M.; Desmond, P.V.; Frye, R.F. Effect of the acute phase response induced by endotoxin administration on the expression and activity of UGT isoforms in rats. *Drug Metab. Lett.* **2008**, *2*, 248–255.
4. Maeng, H.J.; Doan, T.N.K.; Yoon, I.S. Differential regulation of intestinal and hepatic CYP3A by 1alpha,25-dihydroxyvitamin D3: Effects on in vivo oral absorption and disposition of buspirone in rats. *Drug Dev. Res.* **2019**, *80*, 333–342.
